# Stomagenesis versus myogenesis: Parallels in intrinsic and extrinsic regulation of transcription factor mediated specialized cell‐type differentiation in plants and animals

**DOI:** 10.1111/dgd.12282

**Published:** 2016-04-29

**Authors:** Aarthi Putarjunan, Keiko U. Torii

**Affiliations:** ^1^ Department of Biology University of Washington Seattle Washington 98195 USA; ^2^ Howard Hughes Medical Institute University of Washington Seattle Washington 98195 USA

**Keywords:** bHLH proteins, cell‐cycle regulators, cell‐state transition, mitogen activated protein kinase cascade, peptide signaling, receptor kinases

## Abstract

Although the last common unicellular ancestor of plants and animals diverged several billion years ago, and while having developed unique developmental programs that facilitate differentiation and proliferation specific to plant and animal systems, there still exists a high degree of conservation in the logic regulating these developmental processes within these two seemingly diverse kingdoms. Stomatal differentiation in plants involves a series of orchestrated cell division events mediated by a family of closely related bHLH transcription factors (TFs) to create a pair of mature guard cells. These TFs are in turn regulated by a number of upstream signaling components that ultimately function to achieve lineage specific differentiation and organized tissue patterning on the plant epidermis. The logic involved in the specification of the myogenic differentiation program in animals is intriguingly similar to stomatal differentiation in plants: Closely‐related myogenic bHLHs, known as MRFs (Myogenic Regulatory Factors) provide lineage specificity essential for cell‐fate determination. These MRFs, similar to the bHLHs in plants, are regulated by several upstream signaling cascades that succinctly regulate each differentiation step, leading to the production of mature muscle fibers. This review aims at providing a perspective on the emerging parallels in the logic employed by key bHLH transcription factors and their upstream signaling components that function to precisely regulate key cell‐state transition events in the stomatal as well as myogenic cell lineages.

## Graphical abstract

Lineage specific differentiation and organized tissue patterning is central to maintain overall growth and development in all eukaryotes. Plants and animals, despite belonging to vastly divergent kingdoms, use a highly conserved logic to specify organized patterning events in various cell and tissue types. The stomatal differentiation program in plants as well as myogenic differentiation program in animals utilizes a family of closely related bHLH transcription factors and other upstream signaling and cell‐cycle components to specify guard cell and mature muscle cell fate.

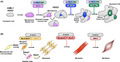

## Introduction

Specification of cell‐fate during development requires the coordinated expression of key transcription factors that regulate lineage specificity as well as cell state transitions. In plants, due to their sessile nature and lack of cell mobility, organized differentiation of functional tissue patterns becomes mandatory in order to survive and maintain the overall fitness of the organism. For example, the vascular system has a well‐defined growth axis that undergoes orchestrated division events, both radially and tangentially, to support plant growth both above and below the ground (root‐shoot system) (Nieminen *et al*. 2015; De Rybel *et al*. 2016).

**Figure 1:**
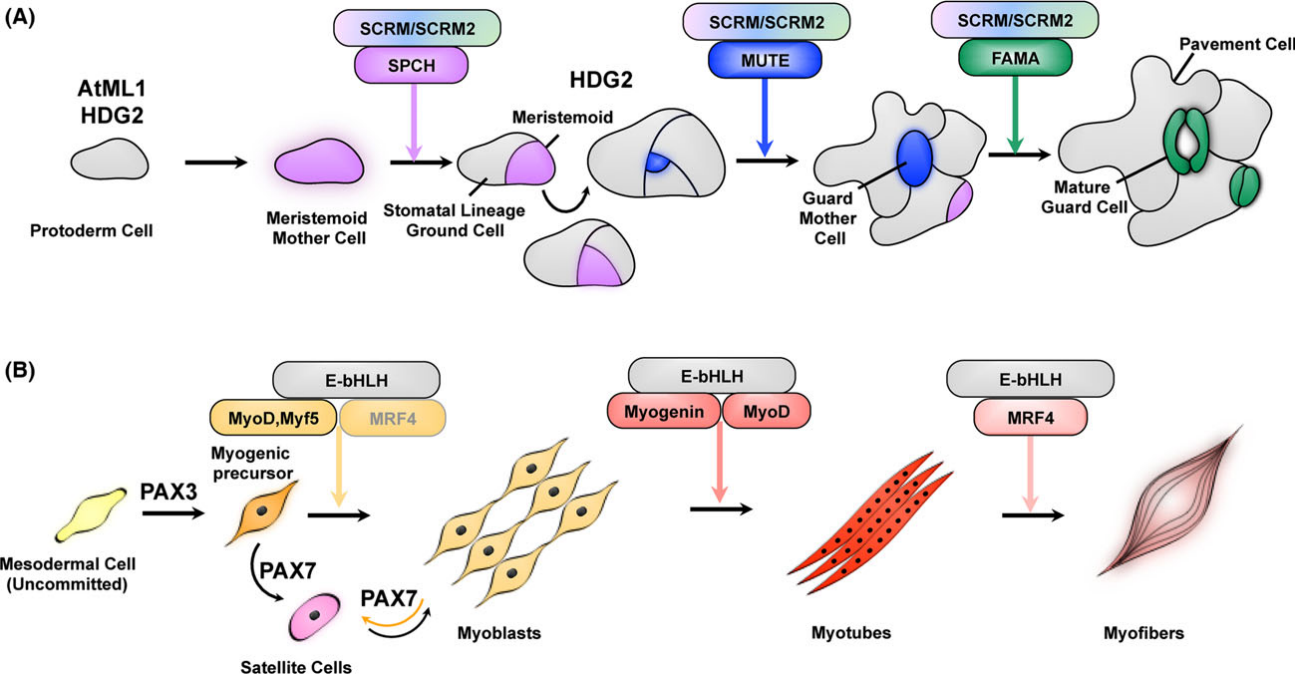
Conserved logic utilized during stomagenesis and myogenesis. (A) Stomatal development pathway in plants. Following the specification of epidermal cell identity by the homeodomain proteins (HD‐ZIP IV), AtML1and HDG2, a subset of protodermal cells form the meristemoid mother cells (MMC). The MMC then undergoes asymmetric division to give rise to a meristemoid, and a larger sister cell called the stomatal‐lineage ground cell (SLGC). The meristemoid undergoes several rounds of asymmetric divisions giving rise to a guard mother cell (GMC), which then undergoes one last round of symmetric division to form a pair of guard cells or mature stomata. Each of these cell state transition events are uniquely regulated by a family of closely related bHLH transcription factors namely: SPCH (that specifies entry into asymmetric division depicted in magenta), MUTE (that specifies GMC fate depicted in blue) and FAMA (that specifies GC fate depicted in green) and their heterodimeric partners SCRM/SCRM2. Entry into the stomatal lineage is mediated by the SPCH•SCRM module, which promotes its own expression. The meristemoid undergoes iterative asymmetric divisions to renew itself and amplify neighboring SLGC cells. The MUTE•SCRM module facilitates the transition from the meristemoid to the guard mother cell, thereby terminating the stem‐cell fate. The FAMA•SCRM module mediates the transition from the guard mother cell to the guard cell. These three distinct modules act in concert to enforce lineage specificity and organized patterning during stomatal differentiation. (B) Myogenic differentiation pathway in animals. The homeodomain proteins PAX3 and PAX7 are required to select myogenic progenitor cells and to regulate the transition between myoblasts and the myogenic satellite stem cells in the quiescent state, respectively. Like in stomagenesis, the myogenic differentiation program is regulated by the MRF family of closely related bHLHs – MyoD, Myf5, Myogenin and MRF4 that heterodimerize with E‐BHLH proteins to promote lineage‐specific cell‐state transitions. MyoD and Myf5 act during the initial steps of differentiation, regulating proliferation and self‐renewal of myoblasts. Myoblasts transition to myocytes, which start to express Myogenin that facilitates myofiber formation. The mature myofibers continue to express the terminal differentiation regulator, MRF4.

**Figure 2:**
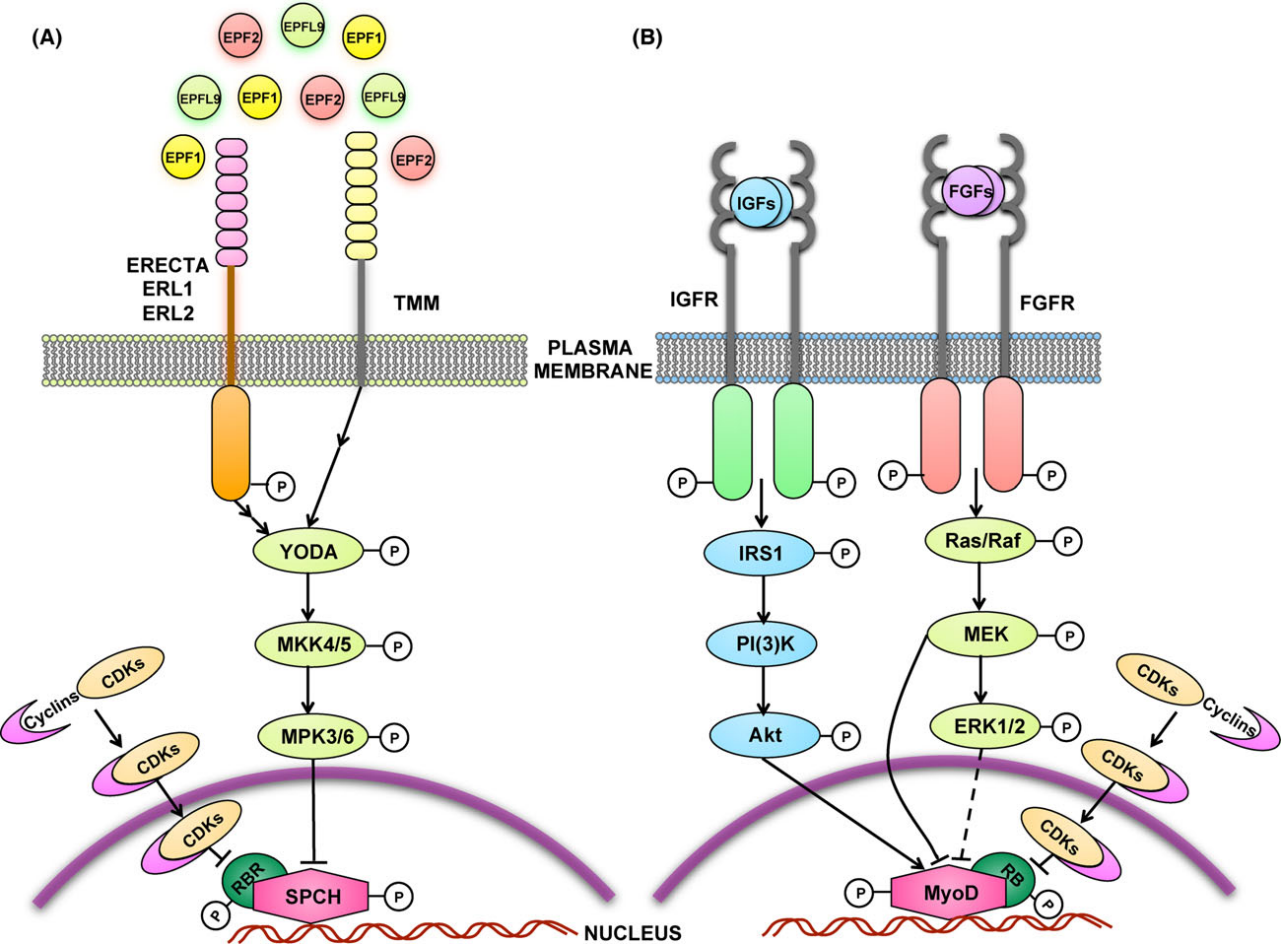
Signaling pathways regulating cell‐fate in stomagenesis and myogenesis. (A) Signal transduction in stomagenesis. Signal transduction in the stomatal development pathway begins with the perception of the EPF family of peptides by the ER family LRR‐RLKs and the receptor like protein TMM. Upon the ligand binding to its receptor, the signal is transduced via the MAPK cascade that includes YDA, MKK4/5 and MPK3/6 downstream of the receptor like kinase/protein. This triggers phosphorylation of SPCH and inhibition of the differentiation pathway. Apart from receptor kinases and MAPKs, cell cycle regulators such as the cyclin dependent kinases (CDKs) and their corresponding cyclins also influence patterning on the epidermis. CDKs are known to phosphorylate RBR that binds to the promoter of SPCH. CDKs influence asymmetric divisions within the stomatal lineage by the regulation of RBR on SPCH transcription. (B) Signal transduction in myogenesis. Signal transduction in the myogenic lineage is achieved by the perception of several growth factor peptides (such as IGFs and FGFs) by their corresponding receptor kinases such as IGFR and FGFR. The IGFs use the PI3K/Akt pathway for signal transduction and this pathway plays a critical role in stimulating myoblast differentiation. FGFs achieve signal transduction via the ERK pathway. Upon ligand binding, the downstream MAPK cascade comprising of a MAPKKK (Ras/Raf), a MAPKK (MEK) and a MAPK (ERK1/2) is activated which triggers a series of phosphorylation events that inhibit myoblast proliferation. Like in plants, cell cycle regulators such as CDKs and their analogous cyclins function to repress MyoD transcription by phosphorylating RB that prevents activation of MyoD transcription.

The plant shoot epidermis is composed of specialized cell types that undergo coordinated division events in a non‐random manner in order to carry out distinct functional roles. They include pavement cells that are crenulated cells protecting inner tissues from UV damage, desiccation, and pathogen entry, and stomata, turgor‐driven valves for efficient gas exchange. The stomatal lineage on the plant epidermis is initiated by a subset of protodermal cells called meristemoid mother cells (MMC) (Fig. 1A) that undergo asymmetric division to produce a meristemoid, and a larger sister cell called the stomatal‐lineage ground cell (SLGC). The MMC and the meristemoid have limited self‐renewing ability and hence undergo proliferation without a stem‐cell niche (Lau & Bergmann 2012). The meristemoid undergoes iterative asymmetric divisions leading to the production of a guard mother cell (GMC), which then executes one last round of symmetric division to give rise to a pair of guard cells (Lau & Bergmann 2012; Pillitteri & Torii 2012; Torii 2015). These cell‐state specific transition events are mediated by a family of closely related highly specialized basic‐helix‐loop‐helix (bHLH) transcription factors namely SPEECHLESS (SPCH), MUTE, and FAMA, and their heterodimeric partner bHLHs, SCREAM (SCRM)/ICE1 and SCRM2 (Fig. 1A) (Lau & Bergmann 2012; Pillitteri & Torii 2012; Torii 2015). Upstream of these transcription factors are several key signaling components: for example: secreted peptides, such as the EPIDERMAL PATTERNING FACTORS (EPFs) that are perceived by the ERECTA Family receptor‐like kinases (ER, ER‐LIKE1 (ERL1), and ERL2) and receptor‐like proteins such as TMM (TOO MANY MOUTHS) that activate the MAPK (Mitogen Activated Protein Kinase) cascade thereby conferring specificity to the functions associated with SPCH, MUTE and FAMA (Fig. 2A) (Pillitteri & Torii 2012; Torii 2015).

The consecutive action of bHLH heterodimers and the upstream kinase signaling components highlight the similarities in the underlying mechanisms regulating stomatal differentiation in plants and the specialized cell‐type differentiation in animals, such as neurogenesis and myogenesis (Pillitteri & Torii 2007; Serna 2009; Matos *et al*. 2014). Recent studies on the regulation of bHLHs by upstream signaling components further emphasize the highly conserved logic utilized by two vastly divergent organisms from different kingdoms in order to achieve similar final outcomes: lineage specific differentiation and organized tissue patterning. In this review, we introduce and compare some of the key molecular components that specify stomatal differentiation (here defined as “stomagenesis”) as well as skeletal muscle differentiation, myogenesis, with specific emphasis on the roles of bHLH proteins and upstream kinases: receptor kinases, MAPKs and cyclin‐dependent kinases (CDKs). Through this comparison, we hope to unravel key underlying mechanisms that govern cell‐type differentiation within the context of multicellular tissue types.

## Master‐regulatory transcription factors driving cell‐state transitions

### Stomagenesis by consecutive action of three bHLH proteins

In the stomatal lineage, *SPCH* is required to initiate the first transition step: i.e. the transition from MMC to the meristemoid (Fig. 1A). This facilitates the first asymmetric entry division into the stomatal lineage. The loss‐of‐function *spch* mutants exhibit a complete loss of stomatal lineage cells, giving rise to an epidermis solely composed of jigsaw‐puzzle‐shaped pavement cells. Ectopic inducible expression of *SPCH* produces excess cell divisions in pavement cells, suggesting that SPCH is not only essential but also sufficient to produce stomatal lineage cells (MacAlister *et al*. 2007; Pillitteri *et al*. 2007). SPCH is also required for the accurate expression of the other bHLH transcription factors in the cascade of events leading to stomatal differentiation. Inhibition of SPCH has been found to primarily affect the asymmetric division associated with MMC and meristemoids (MacAlister *et al*. 2007; Pillitteri *et al*. 2007; Robinson *et al*. 2011). In order to initiate the stomatal lineage, SPCH requires shoot protodermal identity (L1), which is regulated by two homeobox transcription factors AtML1 and HDG2 (Peterson *et al*. 2013; Takada *et al*. 2013). It has been shown that ectopic expression of AtML1/HDG2 is sufficient to induce *SPCH* expression and drive the formation of stomata in non‐epidermal cells (Peterson *et al*. 2013; Takada *et al*. 2013).

Genome wide profiling of SPCH targets using chromatin immunoprecipitation sequencing (ChIP‐seq) revealed that SPCH binds to the promoters of key stomatal lineage regulatory genes such as *SCRMs*,* TMM*,* EPF2*,* ERL2*,* BASL* and *POLAR* (Lau *et al*. 2014). Moreover, SPCH binds to roughly a third of genes in the *Arabidopsis* genome, with the most abundant targets being genes involved in transcription, signaling, response to stimulus and regulation of hormone levels within the plant (Lau *et al*. 2014). This is analogous to the bHLH transcription factors regulating myogenesis in animal systems: *MyoD* (see later section) that binds to numerous target genes and functions as a master regulator of myogenesis by fine‐tuning the overall epigenetic and transcriptional landscape during development (Cao *et al*. 2010).

*MUTE* functions to terminate asymmetric cell division and promote meristemoid to GMC identity within stomatal lineage cells. Loss of *MUTE* results in the failure to transition to the GMC state thereby producing meristemoids that undergo excessive amplifying divisions (Pillitteri *et al*. 2007). Consistent with its lineage specific function, the *MUTE* promoter is not active all the time but specifically turns on in the late‐stage meristemoids that are about to acquire GMC fate (Fig. 1A). *SPCH* and *MUTE* have distinct functional roles, i.e. *SPCH* being involved with the entry into asymmetric divisions and *MUTE* being involved with terminating these divisions thereby promoting GMC fate. It has been shown that *MUTE* does not require its DNA binding residues to promote GMC fate (Davies & Bergmann 2014). This suggests that members of a larger transcriptional complex (that includes *MUTE*) might play a role in specifying the GMC fate. Ectopic overexpression of *MUTE* in the protodermal cells results in an epidermis solely composed of only stomata (Pillitteri *et al*. 2007, 2008).

The onset of *MUTE* expression drives the switch from stem cell state to differentiation. As such, what regulates *MUTE* expression at the precise spatiotemporal resolution is an important, yet unanswered question. SPCH has been shown to bind to the *MUTE* promoter (Lau *et al*. 2014), but it is unclear whether SPCH directly represses *MUTE* expression. In addition, HDG2 has been shown to upregulate *MUTE* promoter activity *in planta*, likely through the epidermal‐specific L1 box (Peterson *et al*. 2013). A more recent study delineated the regulatory region within the *MUTE* promoter, which revealed the presence of a 175 bp region necessary and sufficient for late meristemoid‐specific expression (Mahoney *et al*. 2016).

FAMA controls the final cell state transition event of a single symmetric division of GMCs as well as transition to GC state. Loss‐of‐function *fama* mutants produce excessive symmetric divisions resulting in the characteristic caterpillar shaped cells (Ohashi‐Ito & Bergmann 2006). Overexpression of *FAMA* leads to the development of unpaired guard cells in the epidermis as well as the mesophyll layer, suggesting that FAMA is sufficient to specify GC identity (Ohashi‐Ito & Bergmann 2006). Unlike MUTE that does not require its DNA binding residues to confer lineage specificity, FAMA requires distinct DNA binding motifs to promote GC fate (Davies & Bergmann 2014). FAMA variants lacking their DNA binding residues have been shown to rescue *mute* but not *fama*. This highlights the importance of spatio‐temporal regulation of the three related yet unique bHLHs with different DNA‐binding properties to maintain lineage specific functions associated with the bHLH transcription factors.

FAMA recruits RBR (Retinoblastoma‐Related) through its LxCxE interaction motif to maintain guard cell identity (Lee *et al*. 2014a; Matos *et al*. 2014). The FAMA‐RBR module likely regulates transcription of stomatal lineage genes by recruiting the Polycomb repressive complex PRC2, a conserved complex for repression of gene expression in plants and animals, that associates chromatin modification with stable attainment of GC identity (Matos *et al*. 2014). Transgenic lines expressing a copy of the *FAMA* transgene (i.e. *proFAMA:cFAMA‐GFP* [*FAMA* ^*trans*^]) gives rise to a unique “stoma‐in‐stoma (SIS)” phenotype, causing the mature GC identity to reacquire lineage specific stem‐cell like potential thereby causing it to differentiate within the terminally differentiated guard cell (Lee *et al*. 2014a). This phenotype is similar to that observed when RBR is repressed specifically in *FAMA*‐expressing stomatal precursors of wild type plants using the artificial microRNA line amiR‐RBR (Lee *et al*. 2014b; Matos *et al*. 2014). At the molecular level, the SIS phenotype has been correlated with the disruption in levels of H3K27me3 marks on stomatal lineage genes, that leads to de‐repression of stem‐cell gene expression levels (Lee *et al*. 2014b). The SIS effects by the *FAMA* transgene cannot be recapitulated by extra copies of *SPCH* expressed in the later stomatal precursors (Matos *et al*. 2014), again emphasizing the specific nature of FAMA in maintaining the terminally‐differentiated guard cell state.

### Heterodimeric, integrator bHLHs in stomagenesis

SPCH, MUTE and FAMA are regulated by two additional bHLH proteins SCRM and SCRM2 that specify the sequential action of these TFs in a lineage specific manner (Fig. 1A) (Kanaoka *et al*. 2008). A successive loss of *SCRM* and *SCRM2* recapitulates the *fama*,* mute*, and *spch* mutant phenotypes, indicating that SCRMs are absolutely essential to preserve the functions of SPCH, MUTE, and FAMA in a dosage‐dependent manner (Kanaoka *et al*. 2008). SCRM and SCRM2 heterodimerize with the bHLH transcription factors SPCH, MUTE and FAMA and promote lineage specific stomatal cell state transition events (Kanaoka *et al*. 2008). SPCH directly binds to the *SCRM* promoter and upregulates its initial expression (Lau *et al*. 2014), but both SPCH and SCRM are mutually required to initiate entry of asymmetric divisions of stomatal lineage cells (Kanaoka *et al*. 2008; Horst *et al*. 2015). This is consistent with the observation that *spch* as well as *scrm scrm2* mutants phenocopy one another giving rise to an epidermis composed only of interlocking pavement cells (Kanaoka *et al*. 2008; Horst *et al*. 2015) The dominant gain‐of‐function mutant of *SCRM* called *scrm‐D* (that has stabilized SCRM) produces an epidermis composed only of fully differentiated guard cells, resembling the ectopic *MUTE* overexpression (Kanaoka *et al*. 2008; Pillitteri *et al*. 2011). The *scrm‐D* mutation has been shown to stabilize the SPCH/SCRM heterodimer module thereby causing increased entry into the stomatal lineage pathway (Horst *et al*. 2015).

### Skeletal myogenesis by the consecutive action of bHLH proteins

The sequential use of closely‐related bHLH transcription factors in mediating stomatal development in plants is similar to those involved during skeletal myogenesis in vertebrates (Fig. 1B). The skeletal muscles are primarily derived from the paraxial mesoderm that undergoes gradual segmentation to form repetitive epithelial structures called somites. With the maturation of these somites, the myogenic progenitor cells restrict themselves to the dorso‐lateral region of the somites termed as the dermomyotome. The process of skeletal myogenesis originates in the dermomyotome from where it undergoes lineage specific differentiation events to ultimately give rise to muscle fibers (Le Grand & Rudnicki 2007; Buckingham & Relaix 2015).

Throughout embryogenesis within the developing muscle cells, a progenitor population is maintained by the *Pax* homoedomain transcription factor family genes *Pax3* and *Pax7*. This population of myogenic progenitor cells is also referred to as satellite cells and assumes a quiescent state, being involved in muscle regeneration upon injury (Montarras *et al*. 2013). *Pax3* expression is restricted to the dermomyotome, from where the muscle progenitor cells arise, and is required to select myogenic precursor cells (Fig. 1B) (Goulding *et al*. 1991). Once the precursor cells have been activated to enter the myogenic differentiation pathway, the expression of *Pax3* is downregulated. *Pax7* is expressed in cells surrounding the myofibers and is involved in the specification of satellite cells that assume a quiescent state throughout embryogenesis (Relaix *et al*. 2006). *Pax3* and *Pax7* are repressed when the myogenic cells withdraw from the cell cycle with the onset of *Myogenin* expression (discussed below) (Olguin *et al*. 2007). PAX3 has been shown to interact with Retinoblastoma family proteins using its homeobox domain (Wiggan *et al*. 1998). It has been hypothesized that this interaction mediates myogenic progenitor cell fate specification and cell cycle progression during myogenesis.

Following the activation of myogenic progenitor cells, the myogenic lineage is specified by the action of the MRF family of bHLH transcription factors: *MyoD*,* Myf5* (Myogenic Factor 5), *myogenin* and *MRF4*, which act in an iterative manner to direct cell‐fate in sequential steps leading to myoblast differentiation (Fig. 1B) (Le Grand & Rudnicki 2007; Pillitteri & Torii 2007; Knight & Kothary 2011; Buckingham & Rigby 2014; Matos & Bergmann 2014). Myogenesis originates in the somite where *Myf5*, the first bHLH myogenic regulatory TF is expressed. *Myf5* is expressed prior to *MyoD* in some somites (i.e. the epaxial somite), but *MyoD* expression precedes *Myf5* in the other somites (i.e. the hypaxial somite) (Ott *et al*. 1991; Tajbakhsh *et al*. 1997). *MyoD* is transcribed shortly after the onset of *Myf5*. *MyoD* is required to induce differentiation potential in skeletal myoblast cells (Sabourin *et al*. 1999; Cornelison *et al*. 2000) while *Myf5* is responsible for their maintenance and proliferation rate (Gayraud‐Morel *et al*. 2007; Ustanina *et al*. 2007). Although it has been shown that both *MyoD* and *Myf5* compensate for one another during embryogenesis (Rudnicki *et al*. 1993), they do not robustly compensate for one another in the adult cells (Megeney *et al*. 1996; Gayraud‐Morel *et al*. 2007; Ustanina *et al*. 2007). *Myf5* deficiency results in loss of myoblast amplification and delayed embryonic myogenesis, but recovers partially once *MyoD* expression turns on (Braun *et al*. 1992). Loss of *MyoD* results in amplified self‐renewal rather than progressing through the myogenic differentiation pathway, but they compensate for this loss by enhancing and extending *Myf5* expression (Rudnicki *et al*. 1992; Megeney *et al*. 1996; Gayraud‐Morel *et al*. 2007; Ustanina *et al*. 2007).

Once the progenitor cells enter the myogenic differentiation pathway, *Pax3* regulates the enhancer elements controlling *Myf5* transcription. *Myf5* is regulated transcriptionally by numerous enhancer elements present upstream of the gene, thereby allowing accurate spatiotemporal regulation upon entering the myogenic lineage (Moncaut *et al*. 2013). These enhancer elements have been found to be highly lineage specific: they do not activate other closely related bHLHs that control the myogenic differentiation pathway downstream of *MyoD/Myf5*. This specificity in activation is achieved by Transcription Balancing Sequences (TRABs) that function to coordinate enhancer elements with the right promoters (Carvajal *et al*. 2008). Activation of *MyoD* depends on both *Myf5* and *Pax3* as compound *Myf5(Mrf4)Pax3* mutants do not form skeletal muscles in the trunk and limbs (Tajbakhsh & Cossu 1997).

The relationship between PAX3/7 and the myogenic bHLHs is conceptually analogous to that of the homeodomain proteins (HD‐ZIP IV), AtML1and HDG2, which specifies the epidermal identity, with stomatal bHLHs (Fig. 1A) (Pillitteri & Torii 2012; Peterson *et al*. 2013; Takada *et al*. 2013). Ectopic expression of SPCH, MUTE, or the stabilized version of the SCRM protein: *scrm‐D,* fail to confer stomatal differentiation outside of the L1 layer (Pillitteri *et al*. 2007; Kanaoka *et al*. 2008), suggesting that the stomatal bHLHs need epidermal identity to initiate differentiation events. Consistent with this hypothesis, ectopic AtML1 or HDG2 expression triggers ectopic stomatal differentiation in the internal mesophyll tissue (Peterson *et al*. 2013; Takada *et al*. 2013). HDG2 is expressed specifically in the meristemoids and promotes *MUTE* expression, and hence stomatal differentiation (Peterson *et al*. 2013). Thus, the interaction between the HD‐ZIP IV and bHLH TFs occur at multiple steps within the stomatal differentiation pathway.

The expression of *MyoD* during embryogenesis depends on an enhancer element located 20 kb upstream of the gene (Tapscott 2005). Similar to MUTE in stomagenesis, that is capable of overriding cell fate specification upon ectopic overexpression (Pillitteri *et al*. 2008), ectopic overexpression of *MyoD* in the fibroblast leads to trans‐differentiation of muscle cells (Davis *et al*. 1987). Numerous epigenetic programs have been linked to the regulation of myogenic bHLH transcription factors (Buckingham & Rigby 2014). For example: *MyoD* is known to directly bind to a component of chromatin remodeling complex that actively results in chromatin remodeling and therefore transcriptional activation (Forcales 2012). The binding of *MyoD* is restricted by chromatin accessibility; hence the sites that are open are determined epigenetically in a lineage specific manner. Genome wide ChIP‐Seq profiling of *MyoD* binding sites reveals trends similar to SPCH during stomagenesis: i.e. *MyoD* associates with more than 30 000 target genes. *MyoD* binding results in increased histone acetylation marks in the regions where it binds, emphasizing the functional specificities associated with *MyoD* in establishing the lineage‐specific chromatin accessibility (Cao *et al*. 2010).

The induction of *Myogenin* and *Mrf4* is found to be necessary for the development of myotubes and mature muscle cells in adult tissue types (Parker *et al*. 2003). It has also been demonstrated the *Myf5*:*Myod* double null mice could form skeletal muscles only when MRF4 expression in uncompromised. This indicates that MRF4 is not only a myogenic differentiation factor but also could function as a determination factor in the absence of functional Myf5 and MyoD (Kassar‐Duchossoy *et al*. 2004). *Myogenin* is known to induce anti‐proliferative genes leading to myoblast differentiation and hence cell cycle exit (Singh & Dilworth 2013). *MyoD* induces the expression of *Myogenin*, which in turn represses *Myf5* expression and activates *MRF4* (Deato *et al*. 2008; Singh & Dilworth 2013). The expression of *Mrf4* is known to also turn on early in some populations of cells in the early somites (before *MyoD*) (Patapoutian *et al*. 1995). *Myogenin* knockout mice showed differential expression of myogenic lineage markers: i.e. while there was a reduction in *MRF4* levels, *MyoD* accumulation remained normal (Hasty *et al*. 1993). These results indicate that *MyoD* and *Myf5* can compensate for one another and work upstream of *Myogenin* and *MRF4* thereby regulating myoblasts for terminal differentiation (Fig. 1B). ChIP‐seq analyses of these myogenic bHLHs indicate that they associate with both distinct and overlapping loci within the genome (Blais *et al*. 2005; Soleimani *et al*. 2012). However, in differentiated muscle cells, it has been shown that *MyoD* and *Myf5* show nearly identical binding, and their functional specificity appears to lie in their unique properties of transcriptional activation (Conerly *et al*. 2016) . While high‐resolution ChIP‐seq data are not yet available for MUTE or FAMA, it would be interesting to address whether the stomatal bHLHs share binding sites during the stomatal cell‐state transitions.

### Heterodimeric, integrator bHLHs in myogenesis

Upon activation of the cascade of MRFs (*MyoD*,* Myf5*,* MRF4* and *Myogenin*), these transcription factors form obligate heterodimers with different E‐box bHLH proteins (E proteins) (Fig. 1B) (Blackwell & Weintraub 1990). MyoD binds E2A proteins‐E12/E47, to function, and the association of these E proteins with MyoD influences the phosphorylation status of MyoD (Lassar *et al*. 1991). These E proteins are expressed broadly and are capable of forming heterodimers or homodimers, therefore being able to regulate diverse developmental processes outside of the myogenic lineages. For instance, commitment, maturation, and differentiation of B‐lymphocyte lineages are regulated by the combined dosages of the E proteins, E2A, E2‐2, and HEB (Bain *et al*. 1994; Zhuang *et al*. 1996). Neurogenesis is specified by proneural bHLH proteins, Mash1, Neurogenin, and NeuroD, which again form heterodimers with E12/E47 (Ross *et al*. 2003). E12/E47 also regulate epithelial‐mesenchymal transitions (Perez‐Moreno *et al*. 2001).

The diverse expression and the multi‐faceted functions of these E2 proteins share remarkable parallels with SCRMs in plants. Like E2s, SCRM regulates broader developmental and physiological responses outside of the stomatal differentiation pathway. For instance, SCRM was originally reported as a key upstream regulator of cold tolerance (Chinnusamy *et al*. 2003). SCRM also plays a critical role during seed development, where it preferentially heterodimerizes with a specialized bHLH, ZHOUPI (Denay *et al*. 2014). These findings support the notion that SCRMs act as general integrator of lineage‐specific bHLH transcription factors. Future studies on the identification of genome‐wide downstream target sites of SCRMs as well as understanding the modification of SCRM activities during each cell‐state transition may further highlight the similarities and differences in the action of bHLH proteins during stomagenesis and myogenesis.

## Signaling pathways enforcing cell fate

### Peptide receptor kinases in stomagenesis

Stomatal development in *Arabidopsis* is controlled by a family of Leucine Rich Repeat – Receptor Like Kinases (LRR‐RLK), *ER, ERL1, ERL2*, which act together to inhibit stomatal development (Fig. 2A) (Shpak *et al*. 2005). The *ER‐*family genes act in a synergistic manner to ensure cell‐cell communication and coordinate cell fate so as to ensure proper stomatal density as well as correct positioning of stomatal lineage cells (Shpak *et al*. 2005). Different members of the *ER* family, however, are associated with distinct roles towards determining cell fate: *ER* primarily suppresses entry divisions whereas *ERL1* inhibits GMC differentiation (Lee *et al*. 2012). The upstream ligands of *ER*‐family identified thus far all belong to EPF/EPF‐LIKE (EPFL) of small, secreted cysteine‐rich peptides. *EPF2* is directly induced by *SPCH* and *SCRMs* and restricts entry into stomatal lineages (Hara *et al*. 2007; Hunt & Gray 2009; Lau *et al*. 2014; Horst *et al*. 2015), while EPF1 is expressed in later meristemoids and the GMC to enforce proper stomatal spacing (Hara *et al*. 2007). Consistently, ER primarily perceives the EPF2 peptide and prevents entry divisions whereas ERL1 primarily perceives the EPF1 peptide and regulates spacing divisions at the meristemoid‐GMC step (Lee *et al*. 2012).

TMM, an LRR receptor like protein without a cytoplasmic effector domain acts in concert with the three *ER*‐family genes to regulate cell fate and patterning within the stomatal lineage by forming receptor heteromultimers (Fig. 2A) (Nadeau & Sack 2002; Shpak *et al*. 2005; Lee *et al*. 2012). While the secreted peptide ligands EPF1 and EPF2 act as negative signaling components at distinct steps mediating stomatal differentiation (Hara *et al*. 2007, 2009; Lee *et al*. 2012), Stomagen (also known as EPF‐LIKE9), which is primarily expressed in the mesophyll tissues, is a positive regulator of stomatal differentiation (Kondo *et al*. 2010; Sugano *et al*. 2010). Genetic analysis has revealed that *TMM* is epistatic to *STOMAGEN* implying that both positive and negative signals could act on the same pathway (Kondo *et al*. 2010; Sugano *et al*. 2010). Indeed, recent studies have shown that both EPF2 and Stomagen can directly bind to ER and to TMM, and Stomagen competitively replaces EPF2 binding to ER (Lee *et al*. 2015). TMM shows contrasting effects on stomatal development in different organs, likely due to the availability of different EPFL peptides (Torii 2012).

The immediate downstream targets of ER‐family/TMM receptor complexes are unknown. However, both genetic and biochemical evidence indicates that the downstream canonical MAP kinase cascade is activated upon receptor activation (Bergmann *et al*. 2004; Wang *et al*. 2007; Lee *et al*. 2015). While EPF2 peptide treatment, which inhibits stomatal development, activates downstream MAPKs, Stomagen treatment in contrast, which promotes stomatal development, does not (Lee *et al*. 2015).

### Growth factor receptor kinases in myogenesis

Analogous to plant receptor kinases, animal systems utilize growth factor receptor kinases in order to coordinate cell fate within the myogenic lineage (Fig. 2B). Peptide growth factors, such as FGFs stimulate a receptor tyrosine kinase (FGFR), resulting in the phosphorylation of the receptor that facilitates the adaptor complex formation through the Src homology 2 (SH2) domain containing protein GRB2 (Knight & Kothary 2011). The adaptor protein subsequently interacts with the guanine–nucleotide exchange factor (SOS) on the plasma membrane and activates Ras GTPase that facilitates catalytic exchange of GTP and activation of Ras. Ras‐GTP is then known to bind Raf and the activation of Raf (a MAP kinase kinase kinase: MAPKKK) and the downstream MAPK cascade comprising MAPK/ERK (Extracellular signal‐regulated kinase) Kinase (MEK) and ERK1/2 (Knight & Kothary 2011). Among the different ERK‐inducing growth factors that have been known, FGF remains the best characterized in the context of myoblast proliferation (Nagata *et al*. 2010). FGF is known to alter myoblast proliferation in an ERK independent manner as well (Campbell *et al*. 1995; Jones *et al*. 2001).

Similar to FGFs, Insulin Growth Factors (IGFs) regulate myogenesis using the well‐studied PI3K/Akt protein kinase pathway (Fig. 2B) (Glass 2003; Franke 2008). The PI3K/Akt pathway is activated by the binding of the IGF peptides to their receptor IGFRs that contain a tyrosine kinase domain facilitating autoactivation upon ligand binding. IGF/PI3K/Akt is known to play a critical role in stimulating myoblast differentiation and hypertrophy (Coleman *et al*. 1995; Barton‐Davis *et al*. 1998; Musaro *et al*. 2001). IGF activates all three isoforms of Akt. Among them, the activated Akt 1 and 2 promotes the recruitment of MyoD and histone acetyltransferase to downstream target loci for gene expression (Serra *et al*. 2007). The IGFs/PI3K/Akt signaling pathway is known to act in parallel with the MAPK cascade to regulate differentiation (see below).

### Mitogen activated protein kinases (MAPKs) in stomagenesis and myogenesis

The MAPK cascade functions ubiquitously in both plant and animal systems in governing a variety of cellular processes ranging from cellular immunity to differentiation processes (Xu & Zhang 2015). MAPKs in the stomatal‐lineage cells promote pavement cell differentiation and inhibit stomatal cell initiation (Fig. 2A). Constitutive activation of MAPK cascade components, YODA (YDA) (MAPKKK), MKK4/5 (MAPKKs), and MPK3/6 (MAPKs) results in an epidermis composed of only pavements cells similar to *spch* mutants, whereas loss of function *mpk3mpk6* double mutants recapitulate the *scrm‐D* like phenotype, with the epidermis solely composed of mature guard cells (Bergmann *et al*. 2004; Wang *et al*. 2007). Appearance of the *spch* mutant phenotype when components of the MAPK cascade are constitutively activated is indicative of SPCH being a target of the stomatal MAPK cascade (Bergmann *et al*. 2004; Wang *et al*. 2007). Indeed, SPCH is shown to be phosphorylated by MPK3/6 (Lampard *et al*. 2008). Although a protein microarray study reported MUTE and SCRM also as potential targets of the MAPK phosphorylation pathway, there still exists little evidence verifying this result (Popescu *et al*. 2009). Loss of function YDA, MKK4/5, and MPK3/6 mutants produce severe stomatal clustering (Bergmann *et al*. 2004; Wang *et al*. 2007). Surprisingly, the ectopic expression of the constitutively active forms of MKK7 and 9 driven by the FAMA promoter conferred stomatal clustering (Lampard *et al*. 2009), implying that the MAPK cascade may promote stomatal differentiation at the terminal state.

In myoblasts, MAPK ERK activity can be induced by a number of growth factors (Fig. 2B). While some growth factors, such as FGFs and IGFs, activate ERK to induce or maintain proliferation, PDGF (platelet‐derived growth factor) primarily enhances survival (Milasincic *et al*. 1996; Miralles *et al*. 1998; Pizon & Baldacci 2000; Adi *et al*. 2002). During myogenesis, ERK1/2 is critical for growth factor‐induced proliferation activities and inhibits myoblast differentiation (Volonte *et al*. 2005; Kook *et al*. 2008). The activated MEK1, an MKK acting upstream of ERK1/2, binds to MyoD and represses transcriptional activity (Perry *et al*. 2001). However, this involves a mechanism other than MyoD direct phosphorylation or protein degradation, thus different from the known regulation of SPCH by MAPK cascade (Lampard *et al*. 2008).

The intersection of the MAPK cascade with the myogenic bHLH proteins occurs at multiple levels, directly as seen in protein phosphorylations and indirectly as seen in the epigenetic regulation of *MyoD* target sites. For example, p38 MAPK phosphorylates E47 and promotes MyoD‐E47 heterodimerization, which in turn activates muscle‐specific gene expression (Lluis *et al*. 2005). During muscle regeneration, p38 MAPKs recruit chromatin remodeling complex SWI/SNF to the *MyoD* downstream target sites and cooperatively promote *MyoD* downstream gene expression together with IGF/PI3K/Akt pathway, which recruits histone acetyltransferase to the *MyoD* binding sites (see above). Overall, the emerging theme here is that the MAPK cascade directly influences the activities of the master regulatory bHLH proteins during stomagenesis as well as myogenesis.

## Intersection of cell cycle regulators with master regulatory transcription factors

### Cyclin dependent kinases in stomagenesis

Stomatal development in plants follows a stereotypical sequence of asymmetric and symmetric cell divisions (Fig. 1A) (Bergmann & Sack 2007; Pillitteri & Torii 2012). It is therefore not surprising that cell cycle genes play an important role in regulating stomatal differentiation. Transcriptomic analyses have revealed that promoter activities of the genes encoding Cyclin‐Dependent Kinase, *CDKB2;1,* as well as cyclins *CYCB1;2, CYCA2;2,* and *CYCA2;3,* are highly specific to stomatal lineage cells (Pillitteri *et al*. 2011). *CYCA2;3* and *CYCB1;1* have been associated directly with epidermal patterning (Boudolf *et al*. 2009). Ectopic overexpression of both these genes together leads to the increased division in epidermal cells that mimics the *SPCH* overexpression phenotype, whereas overexpression of these genes separately did not produce any marked difference in phenotype, indicating that CYCA2;3 and CYCB1;1 might be functioning together as a complex to mediate epidermal patterning (Boudolf *et al*. 2009). Some cyclin genes have been known to possess tissue specific roles in epidermal patterning: for example, *CYCD4* controls epidermal patterning specifically in the hypocotyl (Kono *et al*. 2007).

The functions of some cell‐cycle regulators are associated with the symmetric division of GMCs. For instance, loss of function of *CDKB1;1* and *CDKB1;2* results in abnormal single guard cells formation (Boudolf *et al*. 2004; Xie *et al*. 2010). The transcript levels of *CDKA;1 and CDKB;1* are negatively regulated by FAMA (Hachez *et al*. 2011). Interestingly, CDKA;1 plays a role both in the initial asymmetric division and the final symmetric division. A recent report has shown that, like MAPKs, CDKA;1 is capable of phosphorylating SPCH (Yang *et al*. 2015). A deficiency in *CDKA;1* causes defects in both initiation of the stomatal lineage as well terminal GMC differentiation (Weimer *et al*. 2012; Yang *et al*. 2014) CDKA;1 is required for *SPCH* function. Loss of CDKA;1 results in a *spch* mutant phenotype giving rise to only pavement cells (Yang *et al*. 2015). Since CDKA;1 and CDKB;1 are known to phosphorylate RBR, which binds to the *SPCH* promoter, it has been hypothesized that the CDKA;1 promotes asymmetric division within the stomatal lineages via regulation of *RBR* on *SPCH* transcription (Nowack *et al*. 2012; Weimer *et al*. 2012). Collectively, these studies emphasize the interwoven regulation between the stomatal bHLH transcription factors and cell‐cycle machinery for precise coordination of cell‐division sequences and to direct cell‐fate during stomagenesis. Such interactions are also central to myogenesis (see below).

### Cyclin dependent kinases in myogenesis

During myogenesis, CDKs prevent precocious differentiation in proliferating myoblasts. CDK4/Cyclin D and CDK2/Cyclin E have been shown to repress *MyoD* transcription and block differentiation (Rao *et al*. 1994; Rao & Kohtz 1995; Skapek *et al*. 1995, 1996; Guo & Walsh 1997; Saab *et al*. 2006). CDK2/Cyclin E represses MyoD by phosphorylating the Retinoblastoma protein Rb, thereby preventing it from activating *MyoD* transcription (Gu *et al*. 1993; Skapek *et al*. 1996). CDK2/Cyclin E phosphorylates *MyoD* at serine 200 (Kitzmann *et al*. 1999; Reynaud *et al*. 1999; Tintignac *et al*. 2000), which causes ubiquitination and degradation of MyoD during the G1 phase of the cell cycle. CDK4/Cyclin D blocks *MyoD* activity possibly through direct binding, although it still remains unclear what mechanism is utilized in this process (Skapek *et al*. 1996; Zhang *et al*. 1999; Lazaro *et al*. 2002). For myoblasts to differentiate, the repressive activity of CDKs must be removed and the cell cycle needs to be exited.

During differentiation, CDK1, 2 and 6 as well as their Cyclins A, B, and E decrease in expression (Knight & Kothary 2011). In contrast, cyclin D3 levels increase and Cyclin D3s are known to strongly interact with CDK2 and CDK4 (Chu & Lim 2000). Yet, CDK‐Cyclin D3 complexes do not show activity during differentiation, indicating that cyclin D3 might work as part of an inhibitory complex during the process of differentiation (Knight & Kothary 2011). Once the cell cycle CDKs have been repressed, the non‐cell cycle CDKs, CDK 5 and 9, become active and play an important role in the myogenic differentiation process. The dominant negative form of CDK5 inhibits differentiation (Lazaro *et al*. 1997). Similar to CDK5, CDK9 activity also increases upon differentiation and is essential for *in vivo* regeneration after injury (Simone *et al*. 2002; Giacinti *et al*. 2006, 2008). Overexpression of CDK9 results in enhanced differentiation (Simone *et al*. 2002). CDK9/CyclinT2 interact with and phosphorylate MyoD, which may upregulate myogenic transcription (Simone *et al*. 2002; Giacinti *et al*. 2008).

## Perspectives

Although multicellularity evolved independently some billion years ago in the evolutionary history of plants and animals, it is fascinating to observe the highly conserved logic that organisms from two highly diverse kingdoms utilize in order to achieve lineage specificity and organized tissue patterning. The remarkable similarities at the molecular level expand our understanding of how bHLH proteins sequentially specify cell‐state transition and how peptide signal transduction pathways as well as cell‐cycle regulators integrate into the pathway to couple cell fate with cell proliferation and patterning. It is well known that, unlike mammalian cells, the plant cells are totipotent and can readily regenerate (Ikeuchi *et al*. 2013). The study of the FAMA‐RBR module further endorses such an idea, highlighting that plant cells actively repress the expression of master regulatory genes in order to maintain the terminally differentiated state (Lee *et al*. 2014a,b; Matos *et al*. 2014). Both stomatal and myogenic bHLHs are tightly regulated at the chromatin level. Unraveling the exact mechanisms behind such regulations may answer questions fundamental to regeneration. The stomatal development pathway is an excellent model system, using which one can readily study how intrinsic as well as extrinsic cellular components regulate organized differentiation events on the plant epidermis. Moreover, given the lack of cell migration in plants, there exists a permanent trace of all the differentiation events that occurred on the plant epidermis since germination; making it a highly tractable system to understand how a lineage‐specific stem cell responds to diverse developmental cues. Further studies using a repertoire of molecular, biochemical and evolutionary approaches may help unravel how these regulatory circuits fine‐tune patterning processes at a spatio‐temporal resolution.
